# Treating with esketamine nasal, will increase blood pressure?

**DOI:** 10.1192/j.eurpsy.2021.896

**Published:** 2021-08-13

**Authors:** V. Rosello-Molina, F.J. Castells Pons, V. Avellon Juarez, M. Barberan Navalon, C. Domenech Cardona, P. Sastre Portes

**Affiliations:** Mental Health, Hospital Francesc de Borja, Gandia, Spain

**Keywords:** Depressive Disorder, esketamine, treating depression, resistant depression

## Abstract

**Introduction:**

Esketamine had been rised as a potential treatment for Resistant Depression, becoming an alternative for the use of Electroconvulsive Therapy. In Spain since 2020, it has been applied for compassionate use but is not widely used. Although Esketamine is defined safe and effective in preliminary studies, there are common side effects which could reduce it use.

**Objectives:**

Increasing blood pressure has been found commonly in ederly population treated with Esketamine Nasal. Studies showed as very common side effect (10% or more) increasing systolic and diastolic blood pressure which is higher in elderly people. Our aim is to show that esketamine is well tolerated and safe in ederly people without increasing blood pressure, although is combinate with oral antidepressant therapy.

**Methods:**

Presenting female 65-year-old with 4 years of treatment maintaining a moderate-severe symptoms. Althougt numerous pharmacological strategies have been attempted, with optimal time and maximum doses, which have been progressively withdrawing showing lack of efficacy or appearance of adverse effects. Among the drugs used we find; 11 antidepressants, 3 antipsychotics, benzodiazepines and even lithium, without response after 6 weeks of treatment. Futhermore, patient refusal to receive Electro-Convulsive Therapy. Treating with Esketamine nasal and applying the established guidelines.

**Results:**

Assess the response to Esketamine Nasal with Montgomery-Asberg depression scale (MADRS) we found that decrease the initial score in 26 points. Evaluating blood pressure before and after each time with no increased value.

**Conclusions:**

Concluding esketamine is well tolerated and safe in ederly people without increasing blood pressure. These findings and results should be confirmed with futher studies.

